# Addressing Health Disparities: How Having a More Diverse Biomedical Workforce Can Contribute to Addressing Health Disparities in Communities that Are Often Underrepresented in the Healthcare System

**DOI:** 10.3389/bjbs.2025.14973

**Published:** 2025-09-22

**Authors:** Victoria Heath, Claire L. Price

**Affiliations:** ^1^ Health, Social Care and Early Years Group, Welsh Government, Cardiff, United Kingdom; ^2^ Swansea University Medical School, Swansea University, Swansea, United Kingdom

**Keywords:** health disparities, health inequities, workforce, diversity, equity diversity and inclusion

## Abstract

Health disparities that are seen in underserved and underrepresented communities are a pressing issue in healthcare. These disparities are embedded into our society through structural inequalities that lead to poorer health outcomes in those from minoritised communities. In its place as the heart of modern healthcare, the biomedical science workforce has the potential to play a crucial role in mitigating these disparities by fostering greater cultural competence, improving patient outcomes and driving innovative solutions. This study reviewed the current literature on the impact of diversity within the biomedical science workforce on health disparities in underserved communities. The review demonstrated where embedded inequities in healthcare lead to worse health outcomes for underserved communities. These disparities are found across healthcare education, diagnostic processes as well as within research and innovation, and this work uses the COVID-19 Pandemic as an example of where health disparities have significant consequences for the communities impacted. This review demonstrates that a diverse biomedical science workforce can not only contribute to better health outcomes, but also to inclusive research agendas and clinical studies by ensuring that research priorities are more representative of a broader population. A more diverse biomedical science workforce can serve as role models and mentors, inspiring the next-generation of biomedical scientists from underrepresented backgrounds creating a continuous cycle of inclusion and representation, helping to reduce health disparities over time. Therefore, a key strategy in promoting health equity is by increasing diversity in the biomedical science field. After review of current published works, the authors have proposed a list of recommendations that outline steps institutions, professional bodies and policymakers could take to a strategic and sustained commitment to improving biomedical science workforce diversity in an effort to reduce health disparities.

## Introduction

Biomedical science sits at the heart of modern healthcare, with an estimated 95% of clinical pathways requiring access to pathology services, yet its potential to address persistent health disparities remains constrained by a lack of diversity within the field [1–3]. Despite advances in medicine, entire communities continue to be left behind, not because of biology, but because of systemic gaps in representation, research, and relevance [4, 5].

These disparities, marked by unequal health outcomes across population groups, are deeply rooted in structural inequalities such as socioeconomic status, race, geography, and historical exclusion from healthcare systems [5–10].

Increasing representation in biomedical science is more than an ethical goal; it is a scientific necessity. Diverse teams contribute broader perspectives, challenge prevailing assumptions, and lead to more inclusive and impactful research [2, 8, 11, 12]. When scientists bring lived experience and cultural understanding into their work, they help ensure that the questions being asked, and the solutions being developed, are relevant to a wider range of communities [12–14].

A more representative biomedical workforce can help correct these imbalances; expanding access to research participation, improving health outcomes, and reshaping the future of science to be more equitable, inclusive, and responsive. This work will review the currently available literature on health disparities and the impact of diversity within the biomedical science workforce and provide recommendations to support the workforce to rebalance these disparities.

## Understanding Health Disparities

Health disparities refer to the preventable and unjust differences in health outcomes experienced by specific population groups [8]. These disparities are not random, they follow clear patterns tied to social, economic, environmental, and structural factors [6, 8]. People from ethnic minority backgrounds, those living in poverty, and individuals in geographically underserved regions consistently face higher rates of chronic illness, reduced access to preventive services, and shorter life expectancies [6, 15–17]. Examples of this include the United States where the maternal mortality rates in Black Americans are more than triple of other ethnic groups [18].

This has also been demonstrated in the United Kingdom where there is a marked difference in life expectancy at birth between those living in the least and most affluent areas - a striking indicator of how socioeconomic position shapes health trajectories [19, 20]. These differences are further exacerbated by ethnicity: minority ethnic populations are more likely to suffer from conditions such as hypertension, diabetes, and stroke at earlier ages and with worse outcomes [21–24].

It is important to highlight that as health disparities are the result of structural inequities, such as discriminatory housing policies, limited access to quality education and environmental injustice that shape people’s lives long before they enter a clinic or hospital [25].

Within healthcare systems, these disparities are further compounded by implicit bias, communication barriers, and mistrust. Patients from underrepresented backgrounds are more likely to experience misdiagnosis, lower-quality care, and diminished engagement with providers. Language differences, cultural misunderstandings, and a lack of representation among clinical staff often lead to poorer outcomes and decreased patient satisfaction [8, 17, 18].

Biomedical science plays a key role in both reflecting and addressing these disparities. Historically research has excluded the populations most affected by poor health outcomes. Minority participants are frequently underrepresented in clinical trials, while research agendas have tended to prioritize conditions affecting majority populations [26]. This could lead to the development of treatments and diagnostic tools that are less effective, or even inappropriate, for some groups.

## The Role of Cultural Competence

Cultural competence refers to the practice of aiming to create a workforce who have explored and are able to understand how cultural differences may shape how a patient’s values relate to healthcare [27]. Much of the public focus rests on frontline clinicians, however, the impact of biomedical scientists, often working behind the scenes in healthcare, is just as critical in shaping equitable health outcomes. By fostering cultural competence amongst its professionals, biomedical science can aim to help address the structural disparities that are embedded in health.

People’s beliefs about illness, how they seek help, respond to diagnoses, and engage with treatment are shaped by cultural, religious, linguistic, and socioeconomic contexts [28–30]. Biomedical science professionals who are culturally competent are more attuned to these factors and better equipped to ensure that their work supports appropriate, inclusive, and responsive healthcare delivery [29, 31–33].

This can impact on diagnostics where a failure to understand cultural variation in diet, language, or symptom expression can lead to misinterpretation of test results, misdiagnosis, or inappropriate treatment pathways, particularly as minority groups remain underrepresented in many baseline studies used for assay reference ranges [34, 35].

Patients from underrepresented communities are more likely to report negative experiences shaped by assumptions, stereotypes, and poor communication [30]. While biomedical scientists may not always interact directly with patients, their decisions and outputs feed directly into clinical practice. When healthcare professionals are aware of cultural nuance, they can more accurately support diagnosis and treatment, and contribute to a system that respects and responds to patient diversity.

While attempting to embed cultural competence in the workplace, care must be taken to avoid reducing a culture to a set of traits, leading to generalisations or stereotyping, which has been raised as a risk with traditional models [36]. In response, the concept of cultural humility has emerged - placing emphasis on self-reflection, an openness to lifelong learning, and a recognition of systemic power imbalances that affect both care and science [29].

Incorporating cultural humility into biomedical science education means creating a space for professionals to question their own assumptions, engage with diverse lived experiences, and understand the broader social conditions that shape health. It also means recognising the limits of one’s own knowledge and actively involving patients and communities as partners in care and innovation [37–39].

Embedding cultural competence and humility across biomedical roles can form a powerful bridge between science and society. This approach supports the broader imperative to build a biomedical workforce that not only reflects, but also understands and serves, the diverse communities it aims to support.

## Diversity in the Biomedical Workforce

Building a culturally competent biomedical science workforce begins with ensuring that the workforce itself is diverse. Representation across ethnicity, gender, socioeconomic background, disability, and other dimensions of identity is essential for both equity in employment and for improving health outcomes across the population. Diverse teams bring a wider range of perspectives and insights, all of which are critical for understanding and addressing the complex health needs of increasingly diverse societies [40].

Despite growing recognition of the importance of diversity, the biomedical science workforce remains unbalanced. In 2024 The Health and Care Professions Council (HCPC) data showed that 55% of registered biomedical scientists identified as White, with just 18% identifying as Asian and 18% as Black [2]. The HCPC 2024 data demonstrates that 4% of registered biomedical scientists reported having a disability, significantly lower than the wider population across England and Wales which reported 17.7% and 20.1% respectively in the 2021 census [2, 41]. In Northern Ireland and Scotland, 24.3% and 21.4% respectively reported living with a long-term illness or health condition in the 2021 (N. Ireland) and 2022 (Scotland) national census [42, 43].

This lack of representation risks a culture of exclusion in which minority voices are absent not only from the workforce but also from decision-making tables, research priority-setting, and institutional policy development [6, 26, 44].

A diverse workforce also plays a vital role in building trust. Communities that have historically been marginalized or harmed by medical institutions, such as Black communities in the wake of unethical experimentation, or migrant populations who have experienced discrimination in healthcare settings, are more likely to engage with systems where they see themselves represented [30, 45]. Representation in the laboratory, in diagnostics, and in public health roles sends a powerful signal that all communities are valued and understood.

In addition, individuals from diverse backgrounds can bring an intrinsic awareness of the challenges their communities face - from barriers to accessing care, to cultural stigmas surrounding certain conditions and specific patterns of disease [46, 47]. These insights can inform relevant, and effective healthcare solutions. For example, during the COVID-19 pandemic, faith centres were used as vaccination centres in order to improve vaccine uptake in previously under-served communities [48].

Diversity is not just about being present, it is also about who feels supported, heard, and is able to thrive. Tokenistic representation without meaningful inclusion can lead to burnout, isolation, and high turnover [49, 50].

Expanding and retaining diversity in biomedical science therefore requires a comprehensive approach: one that addresses recruitment, but also tackles the systemic barriers that prevent individuals from underrepresented backgrounds from advancing. This includes ensuring equitable access to leadership pathways and career development opportunities. This would support the development of a diverse and inclusive biomedical workforce that is central to achieving an equitable healthcare system.

## Impact on Research and Innovation

The impact of a diverse biomedical science workforce extends beyond the laboratory and into the heart of healthcare delivery. When the professionals designing diagnostics, developing treatments, and interpreting data reflect the populations they serve, the results are more inclusive, relevant, and effective [51, 52].

Historically, biomedical research has been built around majority populations with a lack of representation impacting on effective patient care. Treatments, diagnostic tools, and reference ranges may not perform equally across populations, contributing to misdiagnosis, adverse drug reactions, and suboptimal care. For example, pulse oximeters have been shown to deliver less accurate readings in individuals with darker skin tones, a flaw that remained unchallenged for years due to the lack of diversity in both clinical testing and scientific scrutiny [53–55].

Diverse teams are more likely to ensure that research designs account for biological, environmental, and sociocultural variation [56]. Additionally, they are more likely to prioritize health challenges that disproportionately affect their own communities, such as asthma prevalence in urban Black populations and deprived areas, higher stroke risks among South Asians, or limited access to reproductive care in migrant communities [57–59]. These insights shift the focus of biomedical science toward greater health equity.

Beyond content, diversity strengthens the scientific process itself. Numerous studies have shown that teams with varied backgrounds are more innovative, more rigorous in testing assumptions, and better at solving complex problems [59–61]. In biomedical science, this intellectual diversity is critical to developing the kinds of robust, real-world solutions.

Diverse researchers are more likely to engage in community-based and participatory research, using methods that emphasise collaboration with affected populations and promote mutual trust [62]. This not only improves data quality but can help ensure that research findings are actually implemented in ways that benefit the people they are meant to serve.

Underrepresentation in research does not just affect patients; it influences who benefits from funding, publication opportunities, and academic recognition [63]. Structural inequities in research funding and authorship further marginalise voices, further compounding the issues that disproportionately affect underserved populations by limiting their visibility [64–66].

Addressing these imbalances is essential for the progression of biomedical science. Equitable innovation leads to better technologies, more precise diagnostics, and more effective therapies for the entire patient population.

## Education and Mentorship: A Pipeline Problem

The systemic barriers that affect the biomedical workforce do not start in the workplace, they begin early in the educational journey. The underrepresentation is rooted in structural inequalities that affect access to education, visibility of role models, and the availability of meaningful mentorship [4, 12, 67, 68].

In higher education, attrition rates remain disproportionately high for students on biomedical science programmes [60, 69, 70]. Mentorship is one of the most effective interventions to improve retention, performance, and professional satisfaction among students and early-career professionals from underrepresented groups [29, 71]. Good mentors provide more than academic guidance; they offer psychosocial support, help navigate institutional systems, and serve as tangible proof that success is possible. Mentorship can be the key difference between remaining in a field or seeking employment elsewhere.

Yet, access to mentorship is not equitably distributed. Minority mentors may be overburdened, expected to support large numbers of students or trainees while also carrying the extra labour of diversity-related service and outreach in a ‘cultural’ or ‘time’ tax [72, 73]. This may further embed the current structural inequities that lead to health disparities.

## The Case of COVID-19: A Stress Test

The COVID-19 pandemic served as a global stress test for health systems, exposing long-standing inequalities and demonstrating the life-and-death consequences of structural disparities. Across many developed countries, ethnic minority groups experienced disproportionately high rates of infection, hospitalisation, and death [74, 75].

Frontline roles that carried a higher risk of exposure in transport, social care, retail, and the NHS were disproportionately occupied by people from minority ethnic communities [76]. Compounding this were existing health conditions that have higher prevalence in ethnic minority communities, such as diabetes, hypertension, and cardiovascular disease [77, 78].

Additionally, the pandemic revealed weaknesses in how biomedical science responds to diverse populations. Public health messaging often failed to account for language diversity or cultural nuance. Initial clinical trials for COVID-19 treatments and vaccines underrepresented Black and Asian participants [79, 80]. Diagnostic tools, including pulse oximeters, were shown to be less reliable on darker skin tones, impacting on patients that were more likely to be significantly affected [53–55].

The COVID-19 pandemic highlighted where lack of trust in healthcare was an issue. Communities with historical reasons to distrust healthcare institutions were understandably hesitant about engaging with contact tracing, vaccination, or hospital care [48].

A more diverse biomedical workforce - one equipped with the cultural competence and community ties could be used to bridge gaps in understanding, reassure hesitant populations, and deliver care that felt safe and respectful.

Importantly, the crisis catalysed several rapid interventions, such as community-led vaccination initiatives and culturally tailored public health campaigns [81–83], that demonstrated the value of diverse perspectives and community engagement in health science. These examples offer a blueprint for how biomedical science can be both inclusive and responsive, especially in times of crisis.

The COVID-19 pandemic should be seen as a warning and a call to action. Future public health emergencies, whether pandemics, climate-driven events, or emerging diseases, will demand a workforce that understands the needs of all communities, not just the majority. Investing in diversity, equity, and inclusion within biomedical science is no longer optional, it is a matter of public health preparedness and ethical responsibility.

## The Laboratory Workforce: Often Overlooked, Critically Important

While clinicians and researchers often occupy the spotlight in conversations about healthcare, the laboratory workforce remains one of the most vital (and most under-recognised) pillars of the biomedical system. Biomedical scientists, clinical scientists and laboratory support staff form the backbone of disease detection, monitoring, and surveillance, often without significant public visibility.

The COVID-19 pandemic brought this issue into sharp relief. Laboratory teams were responsible for rapidly developing and scaling up diagnostic tests, tracking viral variants, and informing national and global health responses with significant capacity and reporting issues [84]. In the UK, the Lighthouse Labs network and NHS laboratory services worked tirelessly under immense pressure [85, 86]. Despite this, the contributions of biomedical science professionals were rarely acknowledged in public discourse, and even less so were the disparities within the workforce itself.

Diversity within the laboratory setting is not just a matter of workplace equity, it directly affects the quality and inclusivity of healthcare. A homogenous lab workforce risks blind spots in diagnostic development, data interpretation, and decision-making [87]. For example, reference values in haematology and biochemistry are often derived from narrow population samples, which can lead to misinterpretation of results when applied to individuals from different ethnic backgrounds [88, 89]. A more diverse workforce brings the lived experience and cultural insight needed to question these defaults, advocate for more representative data, and ensure diagnostic accuracy for all patients [90, 91].

Inclusive laboratory environments tend to foster innovation, collaboration, and retention. Diverse teams are better positioned to spot emerging health trends, challenge bias in test development, and ensure that the tools used in clinical decision-making are robust and equitable [6]. These qualities are especially critical during public health emergencies, where rapid, culturally informed responses can save lives.

Despite their importance, many aspiring biomedical scientists from underrepresented backgrounds face significant barriers to entering and advancing in the field. For those who do enter the profession, experiences of microaggressions, cultural exclusion, and inequitable career progression can hinder retention and morale [92].

Addressing these issues requires deliberate investment. Diversity, equity, and inclusion (DEI) must be embedded into laboratory training programmes, professional development opportunities, and workplace cultures [93, 94].

By investing in a more diverse and inclusive laboratory workforce, the biomedical science field can not only improve the quality and relevance of diagnostics, but also ensure that the broader healthcare system is better equipped to serve all communities. Recognition, representation, and inclusion in this often-overlooked corner of healthcare are long overdue—but critically important for advancing health equity.

## Policy and Institutional Interventions

As demonstrated so far in this work, addressing inequity in the biomedical science workforce requires systemic, sustained change at the institutional and policy levels. Policies and initiatives that aim to increase diversity, equity, and inclusion (DEI) must be woven into the fabric of biomedical institutions - from universities and research councils to healthcare organisations and professional bodies. Policies will require measurable goals and clear frameworks for accountability to avoid any changes being short-lived or failing to demonstrate impact.

There are UK models to improve workforce equity that are currently being implemented. The NHS Workforce Race Equality Standard (WRES) tracks disparities in hiring, promotion, and staff experience by ethnicity, holding organisations accountable for progress [51]. Similarly, the Athena Swan Charter, originally focused on gender equality in academia, has expanded to include intersectional approaches to inclusion across disciplines, including biomedical sciences [51]. In the U.S., the Department of Health and Human Services (HHS) Disparities Action Plan provides a comprehensive roadmap for reducing health disparities through inclusive research, workforce development, and community engagement [95].

However, policies must move beyond checklists and reporting. Real change requires action: actively recruiting from underrepresented groups, providing anti-bias and cultural humility training, reforming leadership pipelines, and removing structural barriers to promotion and career advancement. Institutions must be prepared to challenge long-standing practices that have historically favoured homogeneity - whether in hiring committees, grant allocation, or the peer review process. The regulatory body for biomedical scientists, the Health and Care Professions Council, updated standards of proficiency that all biomedical scientists practising in the UK must uphold to place a greater emphasis on equality, diversity and inclusion [96].

Implementation of such policies must be accompanied by transparency, with regular collection and publication of workforce demographic data allows institutions to identify gaps, track progress, while also providing external accountability.

The structural nature of health disparities demands structural responses. When institutions take a proactive, policy-driven approach to inclusion, the biomedical workforce becomes more reflective of society, and better equipped to improve health outcomes for all.

## Recommendations for the Future

To effectively reduce health disparities and ensure that biomedical science is responsive to the needs of all communities, a strategic and sustained commitment to workforce diversity is essential. The previous sections have shown how underrepresentation affects not just who enters the profession, but also what questions are asked, how health data is interpreted, and who benefits from biomedical innovation. The following recommendations outline concrete steps that institutions, policymakers, and professional bodies can take to drive meaningful change. These recommendations are targeted towards the UK biomedical science workforce, but could be replicated in diagnostic laboratory workforces in other countries:

### Early Pipeline Development

Diversity in biomedical science begins long before individuals enter the profession. Outreach programmes targeting primary and secondary schools, particularly in underserved areas, can help build early awareness of biomedical science careers. Initiatives should include hands-on lab experiences, school visits from diverse professionals, and partnerships between educational institutions and healthcare organisations to provide clear, supported pathways into the field.

### Inclusive Education and Curriculum Reform

Biomedical science education must be relevant to the populations it ultimately serves. Academic curricula should embed cultural competence, cultural humility, and an understanding of health disparities from the outset. Teaching should challenge the myth of scientific neutrality and highlight the social and ethical contexts in which biomedical science operates.

### Structured and Culturally Responsive Mentorship

Mentorship is a powerful tool for retention and success, but it must be intentional and inclusive. Institutions should develop structured mentoring programmes that match students and early-career professionals with diverse, trained mentors who can provide both academic guidance and psychosocial support. Mentors should receive training in culturally responsive practices to avoid reinforcing systemic biases.

### Leadership Diversity and Representation

Representation in leadership sends a strong message about an organisation’s values. Institutions must prioritise the promotion of underrepresented individuals into leadership roles and ensure that decision-making bodies reflect the communities they serve. This includes rethinking criteria for advancement, addressing bias in evaluations, and providing leadership development opportunities that are accessible to all.

### Data Transparency and Accountability

Institutional progress on diversity must be measurable. Regular collection and public reporting of demographic data on hiring, promotion, pay equity, and workplace climate are essential for tracking progress and identifying gaps. These data should inform targeted interventions and be reviewed by internal and external stakeholders to ensure accountability.

### Equitable Research and Funding Structures

Funding agencies and research institutions must ensure that their processes do not unintentionally exclude underrepresented researchers or populations. This means supporting community-led research, requiring diversity plans in funding applications, and investing in studies that address health issues in marginalised groups. Peer review panels and grant boards should also be diverse and trained in recognising unconscious bias.

### DEI Integration in Laboratory and Clinical Practice

All biomedical science workplaces (laboratories, diagnostic centres, and public health teams) should integrate DEI principles into hiring, training, and daily operations. Culturally competent workplace practices improve morale, foster innovation, and ensure that the scientific outputs of these environments are more accurate and equitable. Diversity should be seen not as a separate issue but as integral to the function and success of biomedical science.

### Community Engagement and Co-Creation

Health equity cannot be achieved without engaging the communities most affected by disparities. Biomedical institutions should form partnerships with community organisations to co-design research, diagnostics, and interventions. Such collaboration builds trust, enhances relevance, and ensures that science serves real-world needs.

These recommendations are not isolated fixes but part of a broader systems-level change. Achieving a truly diverse biomedical workforce, and the equitable health outcomes it can support, requires consistent effort, leadership commitment, and a willingness to challenge the *status quo*. The future of biomedical science must be inclusive by design, not by exception.

## Conclusion

Health disparities are not inevitable. They are the result of systems and structures that have too often excluded, overlooked, or underserved entire communities. Biomedical science, as a foundation of modern healthcare, holds enormous potential to challenge these inequities. But that potential cannot be fully realised without a workforce that reflects the diversity of the populations it aims to serve.

Throughout this review, the case has been made that diversifying the biomedical workforce is not only a moral imperative but a practical necessity. Representation enhances cultural competence, supports inclusive education, drives innovative research, and strengthens public trust. It ensures that the insights, knowledge, and lived experiences of all communities are valued and embedded within the science that shapes healthcare delivery.

When biomedical science professionals come from a range of ethnic, cultural, socioeconomic, and geographic backgrounds, they bring perspectives that improve the relevance and effectiveness of diagnostics, treatments, and public health strategies. A more inclusive laboratory workforce can identify gaps in diagnostic tools, push for better data, and serve as advocates for equitable care, even behind the scenes.

As global health challenges grow more complex (from pandemics and climate-related illnesses to rising chronic disease) ensuring that biomedical science is equipped to serve all communities is more urgent than ever. A diverse workforce is not a silver bullet, but it is one of the most powerful tools we have for creating a more just and effective health system.

To build a future where everyone has the opportunity for good health, the field of biomedical science must reflect the full spectrum of society - not just in its workforce, but in its values, priorities, and practices. That future begins now.
